# Translation Inhibition Mediated by Interferon-Stimulated Genes during Viral Infections

**DOI:** 10.3390/v16071097

**Published:** 2024-07-08

**Authors:** Alexandria Smart, Orian Gilmer, Neva Caliskan

**Affiliations:** 1 Helmholtz Institute for RNA-Based Infection Research, Helmholtz Centre for Infection Research (HIRI-HZI), Josef-Schneider-Strasse 2, 97080 Würzburg, Germany; alexandria.smart@helmholtz-hiri.de (A.S.); orian.gilmer@helmholtz-hiri.de (O.G.); 2 Regensburg Center for Biochemistry (RCB), University of Regensburg, 93053 Regensburg, Germany

**Keywords:** interferon-stimulated genes, viral RNA, translation, antiviral response, recoding

## Abstract

Viruses often pose a significant threat to the host through the exploitation of cellular machineries for their own benefit. In the context of immune responses, myriad host factors are deployed to target viral RNAs and inhibit viral protein translation, ultimately hampering viral replication. Understanding how “non-self” RNAs interact with the host translation machinery and trigger immune responses would help in the development of treatment strategies for viral infections. In this review, we explore how interferon-stimulated gene products interact with viral RNA and the translation machinery in order to induce either global or targeted translation inhibition.

## 1. Introduction

**Figure 1:**
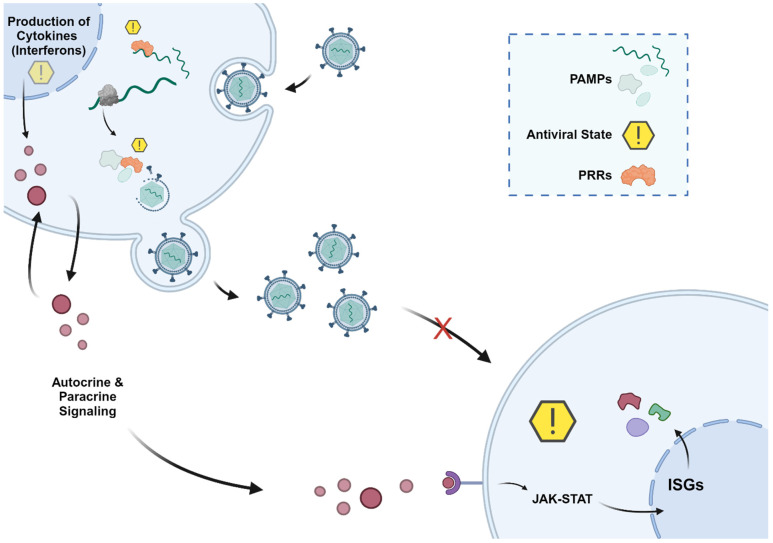
Interferon response triggered by viral infection. Upon viral infection, cells detect specific pathogen-associated molecular patterns (PAMPs) via pattern recognition receptors (PRRs), triggering the initiation of an antiviral response in the infected cell. This process begins with the production of cytokines, notably interferons (IFNs). Released IFNs not only increase the antiviral response in the infected cells but also induce one in neighboring cells through both autocrine and paracrine signaling mechanisms, respectively. IFNs bind to their respective cell surface receptors, initiating signaling cascades primarily via the JAK-STAT pathway or its variants. This activation leads to the engagement of transcription factors capable of promoting the expression of genes governed by interferon-stimulated response elements (ISREs) or gamma-activated sequences. Consequently, ISGs are synthesized, actively contributing to the establishment of the antiviral state within the cell, even preemptively prior to viral infiltration.

Viruses are obligatory intracellular parasites that manipulate and hijack various cellular machineries for their own benefit, frequently posing a significant threat to their host [1]. From entry to egress, they evolved mechanisms to exploit host cellular machinery at every stage of their life cycle. Therefore, virus–host interactions are paramount to the progress of viral replication cycles, especially those involved in the translation of viral RNA (vRNA) into functional viral proteins [2]. Due to the reliance of viruses on host translational mechanisms, host cells can impede viral infection through the activation of the innate immune response by the recognition of pathogen-associated molecular patterns (PAMPs). PAMPs, which usually originate from viral nucleic acids [3] or proteins [4,5], are sensed by pathogen recognition receptors (PRRs). Upon recognition, PPRs induce a signaling cascade, ultimately leading to the production of various cytokines to orchestrate an antiviral immune response (Figure 1).

Among the cytokines secreted upon infection, those from the interferon (IFN) family have been shown to modulate numerous host processes and are well known for their ability to provide a strong first line of defense against invading pathogens [6]. IFNs induce an antiviral state in infected and neighboring cells through autocrine and paracrine signaling pathways [7,8]. One way this occurs is by IFNs binding to cell surface receptors, which initiates a signaling cascade involving the downstream activation of the JAK-STAT pathway. This ultimately leads to the transcriptional upregulation of IFN-stimulated genes (ISGs) [9,10]. However, viruses can also repress the activation of IFN pathways in host cells, thus increasing their chances of evading an antiviral response [11,12,13,14,15].

**Figure 2:**
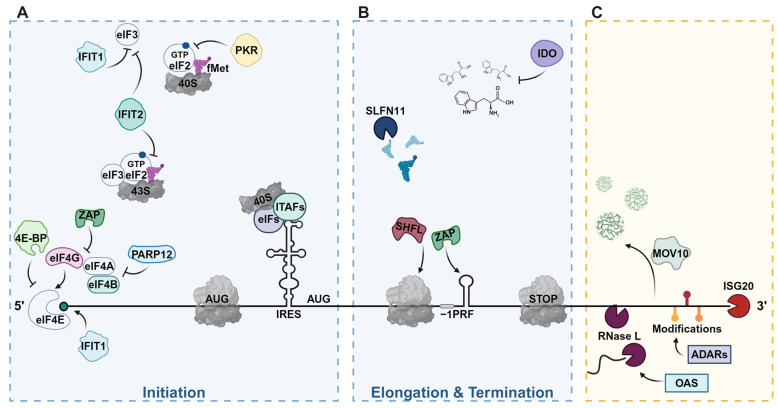
The myriad host interferon responses. Host restriction factors can either inhibit the translation of vRNA or remove vRNA from the host translation machinery. Due to the complex interplay and rapid evolution of virus–host mechanisms, there are multiple ways to restrict viral protein translation during ribosomal initiation (**A**), elongation, and termination (**B**) as well as degradation or localization of vRNAs with exonucleases (**C**). Specific RNA elements (e.g., IRES or –1PRF signal) act as sites for protein binding to regulate individual steps of translation.

Upon infection, IFN-stimulated host restriction factors that slow viral biogenesis can target every phase of the viral replication process [9]. Driven by genetic conflicts with frequently evolving viral counterparts, restriction factors also tend to exhibit signs of rapid evolution [16]. This dynamic is often analogized to the “Red Queen’s interaction”, due to the imperative nature of continual adaptation for both parties. Thus, the interactions of viruses and host factors exist in a perpetual state of adversarial co-evolution [17]. In this review, first, we will examine the direct mechanisms of vRNA translation inhibition mediated by ISG cascades. Second, we will delve into the indirect regulation resulting from the degradation of vRNA (Table 1, Figure 2).

## 2. Overview of Host Translation

Translation is a fundamental biological process conserved across all kingdoms of life. In eukaryotic cells, the translation process involves initiation, elongation, termination, and recycling with initiation occurring through either 5′ cap-dependent or cap-independent mechanisms (Figure 2A) [88,89,90,91]. In cap-dependent processes, the eukaryotic initiation factor (eIF) 4F complex—comprising eIF4A (an RNA helicase), eIF4E (a cap-binding protein), and eIF4G (a scaffolding protein)—recognizes and binds to the 5′ m7G cap of the mRNA [92]. Simultaneously, polyA-binding proteins (PABP) interact with both the polyA tail at the 3′ end and the eIF4G protein, bringing the two ends of the mRNA closer together. This interaction enhances the efficiency of translation and increases the affinity of the eIF4F complex for the 5′ cap [93]. Translation initiation begins with the recruitment of the 43S pre-initiation complex (43S), which includes eIF1, eIF1A, eIF3, eIF5, and a ternary complex consisting of eIF2, guanosine 5′-triphosphate, and methionine initiator transfer RNA (Met-tRNA_i_^Met^), to the 40S small ribosomal subunit via the eIF4F complex, thus forming the 48S initiation complex (48S) (Figure 2A) [94,95]. Canonically, the 48S complex scans the 5′ end of the mRNA, unwinding secondary and tertiary structures aided by the eIF4A RNA helicase. Upon recognition of an initiation codon by the Met-tRNA_i_^Met^ in the ribosomal P site, the 60S ribosomal subunit is recruited, and eIFs are released.

Though initiation in eukaryotes generally occurs in this way, for many viral and some cellular mRNAs, a cap-independent mechanism occurs through an mRNA structure known as the internal ribosome entry site (IRES) (Figure 2A) [96,97]. In high-stress conditions, endogenous IRES initiation can be more prevalent, indicating that IRES-mediated initiation within a cell is likely compensating for cap-dependent initiation especially when cap-dependent initiation is actively being inhibited in response to infection [97]. Though IRESs are diverse in sequence and structure, initiation at an IRES occurs by *cis*-regulatory elements on the mRNA such as RNA binding motifs or modifications as well as by *trans*-regulation such as initiation factors or IRES-transacting factors (ITAFs) [96]. IRES types are classified into four categories based on the regulation required for initiation as well as the type of secondary and tertiary structure elements present [91]. Type I IRESs, such as those in Poliovirus (PV) [98], require all initiation factors except eIF4E and recruit the ribosome for standard 5′ to 3′ scanning for the start codon. Conversely, type II IRESs, such as those found in Encephalomyocarditis virus (EMCV) [99], recruit translation machinery directly to a start site without a scanning step. Type III IRESs, such as the one in the Hepatitis C Virus (HCV) [100], require only eIF2, eIF3, and eIF5 to load a ribosome directly at a start codon but are generally composed of more complex RNA structures such as pseudoknots. Also including pseudoknot and complex higher order structured elements are type IV IRESs, which can be found in Cricket paralysis virus (CrPV) [101]. These IRESs do not require translation initiation factors and can promote translation initiation without a genuine start codon or initiator tRNA and are not limited to 5′UTR regions [91].

Once initiation occurs, elongation involves the 80S ribosome translocating along the mRNA, advancing three nucleotides at a time to synthesize the polypeptide chain using aminoacylated tRNAs in a codon-anticodon dependent manner. Upon encountering a stop codon, eukaryotic release factors (eRFs) facilitate the liberation of the newly synthesized peptide. Finally, the ribosomes disassemble into 40S and 60S subunits, poised for a new round of translation.

During viral infection, both the virus and host require host translation machinery to proliferate. Therefore, mechanisms are employed by the pathogens and the host to target two main components: mRNAs and the ribosome. Viruses have evolved numerous host shutoff strategies to interfere with cellular mRNA translation, such as the interruption of processes related to mRNA biogenesis, the degradation of cellular mRNA, or the inhibition of translation [102]. Conversely, cells utilize comparable strategies to impede viral infection, often through the action of ISGs.

## 3. Targeting Translation

Viral replication relies on the host’s translation machinery, making it a prime target for efficient antiviral defense. Here, we explore the intricate landscape of interferon-stimulated factors (ISFs) that either globally inhibit translation (e.g., PKR, IFIT1/P56, IFIT2/P54 2, 4E-BP, and PARP12) or selectively hinder viral translation (e.g., SHFL, SLFN11, IDO, and ZAP). Understanding these mechanisms provides valuable insights into potential therapeutic interventions and the delicate balance between combating viral infections while preserving essential cellular functions.

Interferon-stimulated factors swiftly induce translational arrest by disrupting ribosome initiation (Figure 2A). Chief among them, Protein Kinase R (PKR) phosphorylates eukaryotic initiation factor 2-alpha (eIF2α), leading to a global translational arrest. This widespread impact affects both viral and cellular mRNAs, highlighting the evolutionary importance of a swift response to viral infection [18,19]. Similarly, interferon-induced proteins with tetratricopeptide repeats (IFITs) IFIT1 and IFIT2 act as inhibitors of initiation by binding to eukaryotic initiation factor 3 (eIF3) [103]. This interaction prevents eIF3 from associating with the 40s ribosomal subunit and impairs the recruitment of the mRNA and ternary complex [39,40,41,42,104]. In the context of IRES initiation, IFIT1 has been shown to directly suppress ribosome-vRNA complex formation both in vivo and in vitro [41]. IFITs are able to act as both a sensor and effector to inhibit translation through binding of the 5′ end of non-self RNA. This binding is sequence non-specific, through the phosphate backbone, and targets mainly 5′-triphosphate ends or 5′ capped ends lacking 2′-O-methylation [105]. The recognition of the 5′ end induces a competition between IFITs and the eIF4F complex for the cap binding. Finally, the IFIT antiviral activity seems to be enhanced when IFITs act as a heterodimer (IFIT1:IFIT2) or trimer (IFIT1:IFIT2:IFIT3) [106].

Importantly, the interferon-stimulated gene 20 (ISG20), discussed in more detail later in this manuscript, has been shown to detect foreign RNA and stimulate IFIT1 upregulation [52]. This adds yet another layer of regulation and demonstrates the complex interplay of signaling in interferon response pathways. A related factor, IFIT2/P54, binds to both eIF3e and eIF3c subunits to destabilize the ternary complex, further impeding the formation of the 48S [48].

Another key player in translational regulation during an interferon response is the eIF4E-Binding Proteins (4E-BPs), which directly competes with eIF4G to sequester eIF4E activity [107]. Mammalian target of rapamycin complex 1 (mTORC1)- and downstream target p70 S6 kinase-mediated phosphorylation of 4E-BPs releases the 4E-BP from eIF4E, resulting in the recruitment of eIF4G to the 5′ cap, thereby allowing the translation initiation to proceed [56,57,58,108]. However, hypophosphorylated 4E-BPs, or more specifically the 4E-BP1 isoform, binds tightly to eIF4E to inhibit cap-dependent translation (Figure 2A) [56,57,58,59,60,61,62]. Furthermore, 4E-BP1-negative cell and mouse models are more sensitive to the antiviral effects of IFN treatment upon infection than their 4E-BP1 positive counterparts [60]. Altogether, interferon-activated mediation of 4E-BP allows for immediate regulation of cap-dependent mRNA translation.

Upon viral infection, Poly-(ADP-Ribose) Polymerase 12 (PARP12) can be recruited to initiation factors to modulate the translation machinery and inhibit translation globally. Co-immunoprecipitation studies have shown that PARP12 directly interacts with eIF4B and eIF4A1, while the long isoform (PARP12L) can also associate with ribosomes [67]. The recruitment of PARP12 is dependent on Zinc Finger (ZnF) domains, which allows it to act as a conformational molecular switch further emphasizing the intricate nature of PARP12’s influence on mRNA translation [69]. Though the global shutdown of translation by interfering with initiation is a swift way to combat viral replication, it can have detrimental effects on the cell. For this reason, hosts have evolved more selective inhibitory mechanisms that target the translation of viral mRNAs specifically.

## 4. A Selective Approach: Targeting Viral-Specific Translation

In contrast to global inhibition of translation, some ISGs have evolved to target viral genomes more selectively with limited effect on the cellular translation machinery (Figure 2B). Since virus and host mRNAs can have significant differences in nucleotide composition, one example of selective translation inhibition exploits the codon-usage bias in viral replication [109]. Upon dephosphorylation, Schlafen 11 (SLFN11) selectively binds tRNAs that are stoichiometrically affected upon infection. Acting as a tRNA endonuclease, SLFN11 dynamically adjusts the tRNA pool, offering a nuanced approach to hinder viral protein synthesis [63,64,65,110]. Another example of altering tRNA pools through an interferon response is by Indoleamine-2,3-dioxygenase (IDO). IDO is involved in the catabolism of tryptophan, an essential amino acid for viral replication. By depleting tryptophan, IDO creates a hostile environment for viral protein translation [66]. Though limiting specific amino acids and tRNAs affect viral protein synthesis and thus slows viral replication, host translation is not entirely unaffected. Accordingly, some host factors have also evolved to interact with specific RNA elements to impact viral translation to achieve even more specialized antiviral responses.

One such host factor is the multifaceted approach of zinc finger antiviral protein (ZAP, also known as PARP13), which represses viral translation at target mRNAs. Though there are four isoforms of ZAP, (ZAP-S, ZAP-M, ZAP-L, and ZAP-XL), ZAP-S and ZAP-L have been studied in most detail [111]. Antiviral and translation inhibitory effects and IFN responsiveness of ZAP variants differ, suggesting these isoforms have evolved to mediate distinct antiviral and cellular functions [26,111]. Initially, researchers thought ZAP was slowing infections by contributing to vRNA decay specifically interacting with G-C-rich regions [22,23,24,27,112]. It was later clarified ZAP can also inhibit the translation of vRNA through blocking initiation [25,113]. ZAP-S can associate with 5′ and 3′ UTRs of viral mRNAs to interfere with the interaction between eIF4A and eIF4G, which is required for translational initiation (Figure 2A) [25]. Beyond interference with initiation, ZAP-S has recently been shown to also modulate –1 programmed ribosomal frameshifting (–1PRF) by interacting with specific SARS coronaviruses SARS-CoV-1 and SARS-CoV-2 RNA elements, while not affecting the frameshifting in host mRNAs [29]. Since –1PRF is essential for the correct expression of viral structural and enzymatic proteins, it is an important means of regulating viral translation levels upon ISG activation (Figure 2B).

Another ISG modulator of –1PRF in viral mRNA translation is Shiftless Antiviral Inhibitor of Ribosomal Frameshifting (Shiftless or SHFL), while its role in antiviral regulation is broad, including interaction with other factors such as PABPC1, LARP1, UPF1, and MOV10 [34,37,83,114,115], SHFL inhibits –1PRF through binding to RNA and the translating ribosome (Figure 2B) [35,116]. Interestingly, though SHFL is a strong –1PRF modulator, its ability to restrict viral replication is not limited to this activity since some of the viruses restricted do not possess –1PRF signals [117,118,119]. There is evidence that SHFL interacts with stalled ribosomes, signaling the recruitment of the eRF1-eRF3 complex to result in premature translation termination and mRNA decay [35,120]. SHFL is able to regulate the translation of numerous positive single-stranded RNA viruses, such as *Flaviviridae* (DENV, HCV, WNVKUN, ZIKV, JEV), *Togaviridae* (CHIKV and SINV), and *Picornaviridae* (EMCV), some DNA viruses such as members of *Herpesviridae* [104], many of the *Retroviridae* family (HIV-1, RSV, HTLV, MMuLV, HIV-2), and *Coronaviridae* (SARS-CoV-2) [35,36,37,38,121,122,123]. The diverse strategies employed by ISGs underscore the adaptability and complexity of host antiviral defenses. From global translational arrests to selective viral translation inhibition, these factors exemplify the precision of the host’s response mechanisms.

## 5. Targeting the Template

Interacting directly with the translation machinery represents an effective way to inhibit viral replication by shutting down the entire protein synthesis pathway. Nevertheless, viral protein synthesis can also be impaired by viral mRNA targeting factors that sequester the mRNA template from translation by (1) physically separating the mRNA from active ribosomes, (2) chemically or structurally modifying the mRNA, or (3) degrading the mRNA (Figure 2C).

In mammalian cells, cytoplasmic membraneless compartments are known to form or expand in response to a viral infection. Due to the high concentration of proteins and RNA, these subcellular compartments are often referred to as ribonucleoprotein (RNP) granules, which comprise processing bodies (PB), stress granules (SG), and antiviral granules [124,125]. These granules share some common characteristics such as the absence of translational activity and the presence of non-translating mRNAs, as well as factors involved in translation inhibition and mRNA decay machinery such as the RNA-induced silencing complex (RISC) and its associated factors [126]. Furthermore, a significant portion of the proteins present in these granules have been identified as playing a pro- and/or antiviral function that overlaps with known ISGs (ADAR, APOBEC3F/G, DCP2, IGF2BP2, MOV10, PARP12, PATL1, XRN1, SMG7, ZAP, etc.) [127]. Thus, relocating vRNA into these granules is one of the ways to repress their expression. Importantly, ADAR, MOV10, PARP12, ZAP, and SHFL co-localize with vRNAs in these RNP granules to inhibit viral replication [85,128,129,130,131,132].

MOV10 is a helicase identified for the first time as a protein preventing infection by Moloney Murine leukemia virus (MMuLV). Since then, MOV10 has been shown to be implicated in the inhibition of many viruses, such as HIV-1, HCV, influenza A virus (IAV), or dengue virus (DENV), but also retroelements [80,81,83,84,85]. It functions as an RNA helicase, which regulates microRNA (miRNA) and mRNA generation, maturation, and degradation through RISC and thereby influences viral cycles at various steps [86]. MOV10 recognizes and sequesters HIV-1 gRNA in P-bodies (Figure 2C) [80], but also inhibits IAV RNP by mediating the vRNA degradation through miRNA pathways [84]. In the case of DENV, MOV10 associates with the antiviral protein SHFL to inhibit translation [83]. One of MOV10 modes of action is to bind the G-rich structures near miRNA recognition elements in order to unfold them with its helicase activity and induce their subsequent translational repression or RNA degradation by recruiting RISC [81]. However, some viruses, such as Hepatitis B virus (HBV) or Enterovirus 71 (EV71), managed to co-opt MOV10 to positively regulate their life cycle [79,82]. In addition, downregulation of MOV10 has been associated with defects in the miRNA machinery, which is required by HCV with miR122 [133]. Conversely, MOV10 overexpression also represses HCV replication through an unexplored mechanism [87].

Other factors associated with miRNA-mediated translation inhibition include the Poly ADP-Ribose Polymerase (PARP) family [134]. Among them, PARP12 and PARP13 (ZAP), can also be localized in cell compartments specialized in translational repression [69,131]. Unlike ZAP, PARP12 still possesses its PARP activity, which appears to be involved in the repression of vRNA translation by ADP-ribosylation of Argonaute 2 (Ago2) [68]. While PARP12 has been described for its antiviral activity by inducing viral protein degradation through MonoADP-Ribosylation (MARylation), it seems that its MARylation activity is also required for translational inhibition [67,69]. The importance of the PARP domain is further emphasized by its presence in ZAP-L. Mutations performed in the ZAP-L PARP domain led to a decrease in its antiviral activity, though as previously described, the antiviral activity of ZAP is not exclusive to the PARP domain [29,135]. One of the ways ZAP (-S and -L) and PARP12 repress translation is by binding to G-C-rich RNA sequences through conserved CCCH tandem zinc fingers. In the case of ZAP, it has been shown to bind as a homodimer before recruiting exonucleases, ultimately leading to the target mRNA degradation [136,137]. Additionally, PARP12 inhibits translation by binding to polysomes [67].

The Adenosine Deaminase Acting on RNA 1 (ADAR1) was implicated in the restriction of multiple viral genomes [78]. ADAR proteins are RNA editing enzymes that deaminate adenosines (A) to produce inosines (I) within double-stranded RNA (Figure 2C) [70]. There are two main isoforms of ADAR1 (p150 and p110) which are able to edit viral genomes. Their expression is under the control of an IFN-inducible promoter due to the IFN-stimulated response element (ISRE) located upstream of the p150 promoter region [138,139]. However, there is evidence indicating that p110 is constitutively expressed [77]. Importantly, it was shown that p150 is responsible for most A-to-I editing events targeting both cellular and viral RNAs [77]. Contrary to p110, which is predominantly nuclear, p150 is also present in the cytoplasm and localizes to RNP granules [76]. ADAR editing can be performed in both a highly selective (hepatitis delta virus—HDV; glutamate receptor subunit GRIA2) or nonselective (hypermutation of Measles virus, MeV genome) manner [78]. Cellular machineries, including ribosomes, recognize A-to-I modifications as A-to-G substitutions [140]. Besides its activities on viral genomes, ADAR is also able to edit cellular RNAs such as miRNAs. Both isoforms interact with Dicer and regulate miRNA maturation, as shown by the reduced expression of miRNAs when ADAR1 is absent [141]. Furthermore, ADAR1 editing of immature miRNAs also affects the translational landscape of cells, usually in a cell-type-dependent manner [71,72]. A-to-I substitutions in miRNA lead to a change in the targeted sequences, and thus a change in the regulation of a specific set of genes. In the end, these modifications have been shown to exhibit either positive or negative regulation depending on the specific context; thus, ADAR editing has a broad range of effects in the context of viral replication [73,78,142].

In eukaryotes, the degradation of vRNA serves as a crucial mechanism for controlling viral infections (Figure 2C). Many ISGs targeting vRNA possess an RNAse activity (ISG20) or activate an RNAse with a broad spectrum of targets (OAS—RNase L). In contrast with XRN1, which degrades RNA in RNP granules after decapping, RNase L and ISG20 do not require the mRNA to go through any such preliminary step [143]. A central player in the IFN antiviral pathway is the cellular endoribonuclease RNase L, activated through the 2′-5′-oligoadenylate synthetase (OAS)/RNase L system. Upon OAS activation, the synthesis of 2′-5′-linked oligoadenylates, a unique ligand for RNase L, occurs. RNase L features ankyrin repeats and a catalytic RNase domain, which mediates cleavage of RNA within single-stranded regions, leading to nonspecific degradation of both viral and host RNAs, including ribosomal RNA. This not only leads to the global downregulation of translation, but also eliminates invasive RNAs [20,21].

While ISG20 was briefly mentioned earlier for its role in activating IFIT1, it has also been shown to regulate over 100 genes, many of which are ISGs [51,52]. Mainly functioning as an RNA exonuclease with broad antiviral properties, recent studies challenge the prevailing mechanism of ISG20 by revealing its ability to target specific structures on the hepatitis B virus (HBV) and suggesting inhibition without viral RNA degradation. Using the vesicular stomatitis virus (VSV) as a model, it was demonstrated that ISG20 interferes with viral replication not by degrading viral RNA but by impairing its translation [53]. This translational control mechanism targets all RNAs originating from ectopically introduced genetic material, collectively defined here as “non-self”, irrespective of their viral or non-viral origins. However, ISG20 does not affect the translation of endogenous mRNA transcripts, suggesting its ability to discriminate between the cell’s own versus foreign genetic material [53,55].

Along with interferon-stimulated genes encoding for proteins, miRNA pathways are prevalent in vRNA silencing and can be found in RNP granules [144]. These miRNAs are generally associated with ISGs that regulate vRNA processing in these subcellular compartments [145,146]. One such pathway is mediated by the RNA interference factor Ago2 [147]. The binding of the miRNA to its target sequences requires a perfect base-pair complementarity to induce the cleavage by Ago2 and subsequent mRNA degradation [144]. Otherwise, the binding of both miRNA and Ago2 to target sites represses the translation of the transcript. Although Ago2 expression has not been shown to be upregulated upon viral infection and interferon stimulation, some Ago2-associated miRNAs are explored in [148,149,150,151,152]. The upregulation of these small non-coding RNAs could be responsible for vRNA silencing induced by Ago2 [148,153,154]. In fact, miRNAs were shown to interfere with the replication of multiple viruses such as HCV, PFV-1, VSV, HIV-1, and SARS-CoV-2 [148,154,155,156]. Furthermore, miRNA binding can change the structure of target mRNAs, which seems to be the case for miR-122 and HCV 5′ UTR region, but also for other mRNAs such as SARS-CoV-2 or the human pseudoknot of CCR5 mRNA [133,157,158,159,160,161]. Overall, miRNAs are responsible for the inhibition of translation in many cellular and viral RNAs [162].

## 6. Concluding Remarks

Although it has been essential to confirm the interactions between host factors and viruses, research focusing on specific virus–host factor interactions may carry an implicit bias, by the assumption that certain ISGs exclusively respond to specific viruses. In fact, ISGs are more likely to respond to multiple viral infections. For example, as ZAP research has progressed, the range of viral species modulated has expanded significantly to include Sindbis virus, MMuLV, HBV, Ebolavirus, IAV, and SARS-CoV-2 [163,164,165,166,167,168]. As with ZAP and other factors discussed in this review, it is likely that many other host-encoded factors have a broader phenotype than initially described, and thus likely inhibit a range of viruses upon infection. However, working with cell models that may not always express similar repertoires of antiviral factors, or probe for only specific interactions, we could be missing key information on host–pathogen interplay. Moving forward, it will become increasingly important to utilize techniques that explore virus–host interactions in a complete and unbiased way such as strategies involving large-scale and genome-wide screens, including interactome and multi-omics studies [36,169,170,171]. As we uncover the large complexity between viruses and hosts at a molecular level, advancements in live-cell and single-molecule RNA imaging also allow researchers to visualize vRNA replication and translation processes in infected cells, as well as track the heterogeneity of viral replication dynamics and antiviral responses [172,173,174,175].

In the interferon field, the rapid coevolution of viruses and hosts can be analogized to an arms race: a fight over the translation machinery. This is accurate in many ways, certainly involving the selective pressure of isoform-specific functions in host factors, while viruses continue to evolve new strategies for evasion [26]. However, recently it has been established that there are host factors that are vitally important for the virus life cycle [171]. Human evolution has benefitted from and depended on viral infection strategies. Human endogenous retroviruses are an integral part of the human genome, resulting from ancestral infections of human germline cells. Viruses have played important roles in human development such as the formation of the placenta, neuroprotective functions, and hormone-dependent organ function [176,177]. Thus, the interplay between viruses and host cells becomes even more complex—instead of an arms race to overcome an enemy, a model of coevolution that vitally depends on one another is perhaps more fitting. As we gain a better understanding of the continued dynamic coevolution between virus and host, we may uncover symbiosis where we originally perceived parasitism.

Deciphering the virus–host interplay will continue to be instrumental not only for identifying potential therapeutic targets but also in characterizing general insights into the dynamics of translation. For the development of new RNA-based therapeutics, future work is imperative to advance the understanding of how RNA elements modulate translation efficiency and accuracy in the context of immune responses.

## Figures and Tables

**Table 1:** Host restriction factors and their mechanisms.

ISG	Target (RNA/TM)	Specific Target	Mechanism of Action	References
Protein Kinase R	Translation machinery	eIF2α	Phosphorylation	[18,19]
2′-5′-Oligoadenylate Synthetase (OAS)	RNA (both viral and host)	Activation of RNase L	RNA degradation (including rRNA—translation inhibition)	[20,21]
Zinc finger antiviral protein (ZAP) (PARP13, ZC3HAV1)	Both	eIF4A, mRNA	Initiation inhibition, mRNA decay, –1PRF modulator	[22,23,24,25,26,27,28,29,30]
TRIM25	Both	vRNA and ZAP	Ubiquitination of eIFs ZAP	[28,30,31,32,33]
Shiftless Antiviral Inhibitor of Ribosomal Frameshifting (SHFL) (RyDEN, IRAV, C19ORF66)	Both	mRNA, ribosome	–1PRF modulator, antiviral factor	[28,34,35,36,37,38]
IFN-induced protein with tetratricopeptide repeats 1 (IFIT1) (P56 or ISG56) IFIT5 is paralog	Translation machinery	eIF3e, ternary complex, eEF1A, PKR	Initiation inhibition of vRNA with non-2′O-methylated 5′ cap	[39,40,41,42,43,44,45,46,47]
IFN-induced protein with tetratricopeptide repeats 2 (IFIT2) (P54 or ISG54)	Translation machinery	eIF3c, eIF3e IFIT1	Destabilizing ternary complex, inhibit 48S pre-initiation complex	[47,48,49]
IFIT3	Both	PPP-RNA, IFIT1	Binds PPP-RNA, associates with IFIT1	[43,44,47]
ISG20	RNA (Upregulation of IFIT1)	vRNA, IFIT1	3′-5′ exonuclease, distinguishes between self and non-self RNA, upregulates other ISGs, IFIT1	[50,51,52,53,54,55]
Eukaryotic Initiation Factor 4E-Binding Protein (4E-BP)	Translation machinery	eIF4E	Initiation inhibition	[56,57,58,59,60,61,62]
Schlafen 11 (SLFN11)	Translation machinery	tRNA	tRNA cleavage by codon-bias discrimination	[63,64,65]
Indoleamine-2,3- dioxygenase (IDO)	Translation machinery	Trp metabolism	Amino acid starvation	[66]
PARP12	Both	Initiation factors ADP-ribosylation of Ago2	Inhibition of viral translation, immunomodulation	[28,67,68,69]
Adenosine Deaminase Acting on RNA 1 (ADAR1)	RNA	miRNA, vRNA	dsRNA recognition, vRNA modification, modulation through miRNA pathway	[70,71,72,73,74,75,76,77,78]
MOV10	RNA		Modulation through miRNA pathway, RNP granules relocalization RNA structure unfolding	[79,80,81,82,83,84,85,86,87]
